# Evaluation of the Association Between Medicare Eligibility and Excess Deaths During the COVID-19 Pandemic in the US

**DOI:** 10.1001/jamahealthforum.2021.2861

**Published:** 2021-09-24

**Authors:** Jacob Wallace, Anthony Lollo, Chima D. Ndumele

**Affiliations:** 1 Department of Health Policy and Management, Yale School of Public Health, New Haven, Connecticut

## Abstract

This cross-sectional regression discontinuity analysis compares deaths slightly younger and older than 65 years to examine the relationship between access to health insurance coverage and COVID-19 mortality.

## Introduction

In the US, COVID-19 is responsible for hundreds of thousands of excess deaths,^1^ which has led to considerable interest in strategies to mitigate its impact.^2^ Policy observers hypothesize that access to health insurance could improve COVID-19 survival by facilitating earlier access to testing and treatment.^3^ Concerningly, however, COVID-19 also led to a crisis of coverage in the US because rising unemployment led to many US adults losing their employer-sponsored health insurance coverage.^4^ This study compared deaths among individuals slightly younger and older than 65 years—when most US adults become eligible for Medicare^5^—to examine the relationship between access to health insurance coverage (vs no insurance) and COVID-19 mortality.

## Methods

The Yale University institutional review board reviewed the study and deemed it exempt because we used only retrospective deidentified data; informed consent was waived according to the Regulations for the Protection of Human Subjects (45 CFR §46). The study followed the Strengthening the Reporting of Observational Studies in Epidemiology (STROBE) reporting guideline.

Detailed mortality data for 2015 to 2020 was obtained from Datavant, an organization that augments Social Security Administration death master files with information from newspapers, funeral homes, and memorials to construct an individual-level database of more than 80% of US deaths annually. For each record, the data indicate the month of death and age of the deceased individual in months, allowing us to count deaths in a narrow band around age 65 years. We obtained death counts from the National Center for Health Statistics (NCHS; US Centers for Disease Control and Prevention).^6^

We used a regression discontinuity design, a common method in health care research, to assess whether the death count changed sharply at age 65 years (Medicare eligibility age) between March and December 2020 and for the same span of months in 2015 to 2019 for comparison. We fit a model of the number of deaths by age in quarters with a dummy variable for deaths occurring to persons 65 years or older. The dummy variable captured discontinuities (ie, jumps) in the death count associated with Medicare eligibility. A discontinuity at age 65 years in 2020, but not years prior, would suggest that Medicare eligibility affected COVID-19 mortality.

Statistical analysis was conducted using 2-tailed tests with Huber-White robust SEs to assess statistical significance, defined as *P* < .05. We also assessed the percent increase in deaths for 65- to 74-year-olds by calendar month in 2020 vs calendar month in 2015 to 2019 in the Datavant data and compared it with NCHS data. We reported the average percent increase during the pandemic period and plotted the percent increases by month. Data analyses were performed from December 14, 2020, to July 20, 2021, using Stata, version 16 (StataCorp LLC) and Python, version 3.8 (Python Software Foundation).

## Results

**Figure 1.**
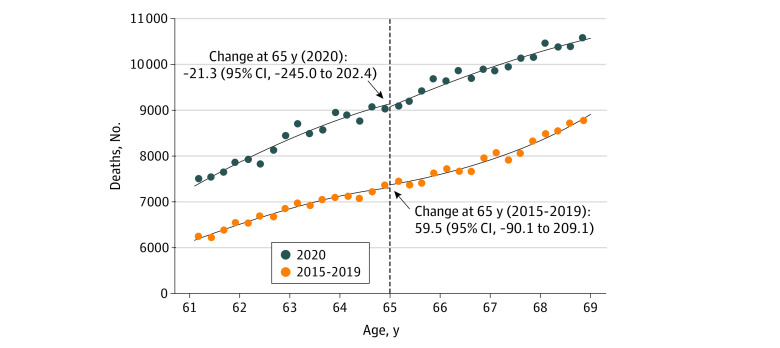
Medicare-Related Discontinuities in Death Counts From March to December, by Time Period For each period (ie, 2015-2019 and 2020), the number of deaths reported in the Datavant data is plotted according to age in quarters at the time of death. For the period 2015 to 2019, points represent the unadjusted mean number of deaths across the 5 years. For illustrative purposes, lines of best fit to the death counts are plotted for 2020 (blue) and 2015 to 2019 (orange); the black dashed line denotes Medicare eligibility age (65 y).

**Figure 2.**
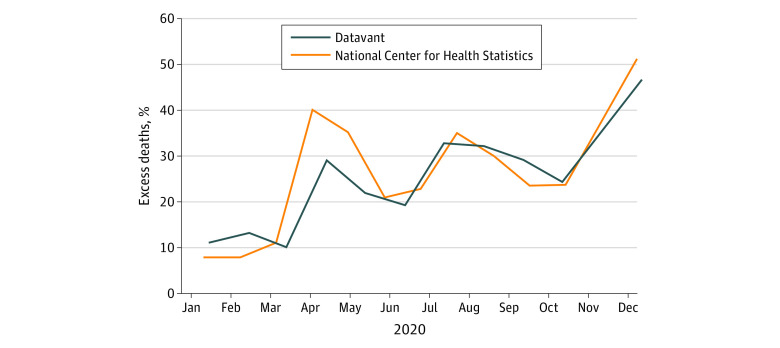
Monthly Excess Deaths From March to December 2020 for 65- to 74-Year-Olds, by Source Unadjusted monthly death counts in 2020 are compared with the mean of the unadjusted death counts in the same month during 2015 to 2019. Datavant data death counts were per calendar month; National Center for Health Statistics data were per week and were aggregated into 4-week periods for comparisons.

This study examined 291 837 deaths among individuals 61 to 69 years old (116 806 [40.0%] women; 175 031 [60.0%] men) between March and December 2020. We did not find evidence of a discontinuity in the number of deaths at age 65 years during the 2020 study period (−21.3; 95% CI, −245.0 to 202.4; *P* = .85) nor during the 5 years prior (59.5; 95% CI, −90.1 to 209.1; *P* = .42; Figure 1). The results were similar when we narrowed the bandwidth around age 65 years or altered the functional form of the relationship between age and death counts (eMethods in the Supplement). The percent change in death counts from March to December 2020 relative to the same months in 2015 to 2019 was 28.1% in the Datavant data and 30.6% in the NCHS data (Figure 2).

## Discussion

Eligibility for Medicare at age 65 years, which has been associated with an immediate and substantial reduction in the uninsurance rate and improvements in measures of access to care,^5^ was not associated with mortality during the COVID-19 pandemic. These null findings may reflect the influence of state and federal policies that directed payments to hospitals for COVID-19 treatment and eliminated cost sharing for COVID-19 testing, both of which aimed to broaden access to COVID-19 testing and treatment.^3^

A serious limitation of this study was that the mortality data, although detailed and recent, did not capture all deaths in the US (only approximately 80%) and did not include the cause of death nor the patient’s race or ethnicity. However, the excess death counts in the present study’s data were similar to those of other reliable sources. This cross-sectional regression discontinuity analysis found that entry into Medicare was not associated with the likelihood of death during the COVID-19 pandemic.

## Notes

Supplement. **eMethods.** Robustness Tests
