# Supplementary material for: Evaluation of the Association Between Medicare Eligibility and Excess Deaths During the COVID-19 Pandemic in the US
Source: JAMA Health Forum. 2021 Sep 24;2(9):e212861. doi: 10.1001/jamahealthforum.2021.2861 (PMC8796977; doi:10.1001/jamahealthforum.2021.2861)
Supplement: Supplement. — eMethods. Robustness Tests [file jamahealthforum-e212861-s001.pdf]

Supplemental Online Content

Wallace J, Lollo A, Ndumele CD. Evaluation of the association between Medicare eligibility and excess deaths during the COVID-19 pandemic in the US. *JAMA Health Forum*. 2021;2(9):e212861. doi:10.1001/jamahealthforum.2021.2861

eMethods. Robustness Tests

This supplemental material has been provided by the authors to give readers additional information about their work.

© 2021 Wallace J, et al. *[JAMA Health Forum].*

eMethods. Robustness Tests

Our preferred specification is a regression discontinuity (RD) design with a quadratic age trend and a 4 year bandwidth around age 65. However, as is common in RD, we assess the sensitivity of our primary results to alternative bandwidths and statistical models. The table below reports primary results alongside alternative specifications. Each column indicates a different approach to specifying the bandwidth around the discontinuity at age 65. The rows are split by time periods, with each of the models (i.e., “Linear”, “Quadratic”, and “Local Linear”) indicating a different approach to modeling the age trend of death counts in our data.

Model	2 year bandwidth around age 65	4 year bandwidth around age 65	6 year bandwidth around age 65	Optimal bandwidth
**Time Period: March 1 2020 – December 31 2020**				
Linear	11.5 (-204.5, 227.6)	-74.4 (-265.2, 116.4)	-90.9 (-260.8, 78.9)	-
Quadratic	-197.3 (-478.1, 83.5)	-21.3 (-245.0, 202.4)	-8.0 (-248.4, 232.3)	-
Local Linear	-	-	-	-91.2 (-735.7, 449.7)
**Time Period: March 1 2015 – December 31 2019**				
Linear	5.0 (-157.8, 167.9)	-176.0 (-298.6, -53.4)	-144.0 (-236.7, -51.3)	-
Quadratic	-16.9 (-189.4, 155.6)	59.5 (-90.1, 209.1)	-133.4 (-276.4, 9.6)	-
Local Linear	-	-	-	-4.2 (-297.6, 339.3)

The table above reports the sensitivity of regression discontinuity results to alterations of the statistical model, including changes in our bandwidth and differences in functional form. Robust (i.e., "Local Linear") estimates rely on the Calonico, Cattaneo, and Titiunik (2014) package,[1] which uses a data-driven process to select the optimal bandwidth and construct bias-corrected confidence intervals.

Below we report a second set of robustness tests, where we alter the time period or the dependent variable. The estimates in the table below are based on our preferred specification, a quadratic age trend (allowed to vary on both sides of the discontinuity) with a 4-year bandwidth.

Model	Raw deaths, No.	Excess deaths, %	Excess Male Deaths, %	Excess Female Deaths, %
**Time Period: March 1 2020 – December 31 2020**				
Quadratic	-21.3 (-245.0, 202.4)	-1.2 (-5.2, 2.8)	-1.2 (-4.0, 1.6)	-1.3 (-9.2, 6.7)
**Time Period: May 1 2020 – December 31 2020**				
Quadratic	-70.6 (-301.0, 159.8)	-2.4 (-6.7, 1.9)	-1.8 (-5.1, 1.4)	-3.2 (-12.7, 6.2)
**Time Period: November 1 2020 – December 31 2020**				
Quadratic	61.2 (-100.2, 222.6)	-3.0 (-9.1, 3.0)	1.0 (-8.4, 10.5)	-9.4 (-21.5, 2.7)

The percent excess deaths (overall and by sex) are calculated by comparing overall (or sex-specific) death rates in March and December 2020 relative to the same months in 2015-2019.

References

1. Calonico S, Cattaneo MD, Titiunik R. Robust nonparametric confidence intervals for regression-discontinuity designs. *Econometrica*. 2014;82(6):2295-2326.

© 2021 Wallace J, et al. *[JAMA Health Forum].*
